# Complete Genomic Characterization of Lumpy Skin Disease Virus Isolates from Beef Cattle in Lopburi Province, Central Thailand, during 2021–2022

**DOI:** 10.3390/vetsci11010010

**Published:** 2023-12-22

**Authors:** Nutthakarn Suwankitwat, Taweewat Deemagarn, Kultyarat Bhakha, Tapanut Songkasupa, Ratchaneekorn Vitoonpong, Pannaporn Trakunjaroonkit, Sureenipa Rodphol, Bandit Nuansrichay, Lerdchai Chintapitaksakul, Khanin Wongsarattanasin, Oh-Kyu Kwon, Hae-Eun Kang, Yeun-Kyung Shin

**Affiliations:** 1 National Institute of Animal Health, Department of Livestock Development, Bangkok 10900, Thailand; deemagarn@hotmail.com (T.D.); kultyarat@gmail.com (K.B.); tapanut.s@dld.go.th (T.S.); vrashaneekorn@gmail.com (R.V.); pannaporn.tr@ku.th (P.T.); traehh626@gmail.com (S.R.); bundit.nu@hotmail.com (B.N.); lerdchai@gmail.com (L.C.); 2 Animal Health Development Group, Lopburi Provincial Livestock Office, Department of Livestock Development, Lopburi 15000, Thailand; khanin.vet71@gmail.com; 3 Animal and Plant Quarantine Agency, Ministry of Agriculture, Food and Rural Affairs, Gimcheon-si 39660, Gyeongsangbuk-do, Republic of Korea; kwonok59@korea.kr (O.-K.K.); kanghe@korea.kr (H.-E.K.)

**Keywords:** lumpy skin disease virus, mutations, live-attenuated LSDV vaccines, Lopburi, Thailand

## Abstract

**Simple Summary:**

The study focuses on the genetic characteristics of the lumpy skin disease virus (LSDV) from non-vaccinated beef cattle with LSD symptoms that lived in areas that had received live-attenuated vaccines in Lopburi, Thailand. Four skin positive samples through real-time PCR underwent virus isolation on cell cultures, followed by whole genome sequencing. The phylogenetic and mutation analyses were used to compare sequences between four LSDV samples from Lopburi with the LSDV/Thailand/YST/2021 strain (original strain in Thailand). In conclusion, four LSDVs in Lopburi during 2021–2022 were recombinant strains, clade 2.5, same as the LSDV/Thailand/YST/2021 strain. However, the viruses had mutations of open reading frame (ORF) 023 with significant amino acid deletion in two samples from Chai Badan district and of the untranslated region between ORF022 and ORF023 in two samples from Lam Sothi district.

**Abstract:**

Lumpy skin disease (LSD) is a viral infection that impacts the cattle industry. The most efficient approach to prevent disease involves the utilization of live-attenuated LSD vaccines (LAVs), which stands out as the most successful method. However, LAVs might be subjected to changes to their genomes during replication that increase viral infectivity or virulence. The objective of this study was to monitor alterations in the genetic characteristics of the lumpy skin disease virus (LSDV) in beef cattle following the administration of LAVs in Lopburi Province of Central Thailand. A total of four skin samples from LSD cases were collected from non-vaccinated animals that exhibited LSD clinical symptoms from two distinct districts, spanning three subdistricts within the region. The samples of cattle were analyzed using real-time PCR targeting the LSDV074 *p32* gene, the virus was isolated, and the entire genome sequences were evaluated through a single nucleotide polymorphisms (SNPs) analysis, and phylogenetic trees were assembled. The investigations revealed that LSDVs from two isolates from Chai Badan district exhibited significant mutations in the open reading frame (ORF) 023 putative protein, while another two isolates from Lam Sonthi district had a change in the untranslated region (UTR). For a result, the most proficient disease diagnosis and control should be evaluated on viral genetics on a regular basis.

## 1. Introduction

Lumpy skin disease (LSD), caused by the lumpy skin disease virus (LSDV), is a highly contagious viral infection primarily affecting cattle and buffaloes, which poses a severe threat to cattle globally. Compared to other ruminants, cattle are more sensitive and susceptible to the disease [1]. In addition, LSD was reported in wildlife such as giraffe in a Vietnamese zoo [2], and gazelles in India [3]. This disease is characterized by the development of characteristic skin nodules, a fever, and a range of systemic symptoms such as peripheral lymph node enlargement and decreased milk production. Severe cases can lead to lameness, emaciation, and even death [4]. Notably, the disease has a high rate of morbidity, but the rate of mortality is relatively low. It has been reported that morbidity and mortality rates in Nizwa and Sohar, Sultanate of Oman, were 29.7 and 26.3%, and 13.6 and 15.4%, respectively [5]. LSDV is primarily transmitted mainly through blood-feeding arthropod vectors (e.g., stable flies, mosquitoes, midges, horse flies, wasps, and ticks). Additionally, transmission can occur mechanically through fomites [6]. It can also be acquired through parenteral inoculation, or exposure to droplets or aerosols on broken skin or mucous membranes [6,7,8,9,10]. It can also spread through direct and indirect contact between infected and susceptible animals. Transporting live cattle or buffaloes has been linked to the long-distance spread of LSDV. Most of these outbreaks occur in the summer, when there are more potential vector species [9,11]. Historically, LSDV was mainly confined to Africa, but it has since spread rapidly and widely to other parts of the world, including the Middle East, Europe, and Asia [12]. LSD has substantial economic implications, as it can lead to significant losses in milk and meat production. Trade restrictions imposed on regions with LSD outbreaks can further exacerbate economic challenges [13]. The disease’s expansion beyond its traditional range has raised significant concerns in global cattle farming. During the course of an outbreak, a commonly advised control measure is the prevention of all susceptible animals exposed to the infection, or at least those exhibiting clinical signs. But the most efficacious strategy for disease control is widespread bovine immunization with live-attenuated vaccines (LAVs). Immunization stands as the sole method to curb infection transmission in both endemic and newly impacted areas. However, during outbreaks, choosing the optimal vaccine poses a significant challenge. The strengths and weaknesses of various live-attenuated vaccination approaches and associated risks were described previously [14]. 

LSDV is a large, double-stranded DNA virus with a 151-kilobase pair (kbp) belonging to the *Capripoxvirus* genus within the *Poxviridae* family. The LSDV genome contains 156 open reading frames (ORFs) [15]. The WOAH recommended ORF074 encoding intracellular mature virion envelope protein P32 for LSDV detection by real-time PCR to determine viral genetic loads in clinical samples [16]. In recent times, recombinant vaccine strains were reported in Russia [17], China [18], Myanmar [19], and Vietnam [20]. These novel strains were most likely the result of a spillover from animals vaccinated with the Lumpivax vaccine (Kevevapi, Kenya), which was widely used in Kazakhstan shortly before the emergence of the vaccine-like strains in Russia [21]. A novel live-attenuated LSD vaccine, Lumpi-ProVac*^Ind^*, has been successfully developed using an Indian field strain isolated from cattle in 2019. In comparison to field/virulent LSDV strains, the vaccine strains, including the Indian vaccine strain, contain a 12 bp insertion in their GPCR gene [22]. Historically, the vaccines have not undergone thorough quality inspections. Hence, it is crucial to assess and scrutinize them thoroughly before their implementation. Quality adherence to good manufacturing practices (GMPs) is crucial for cattle welfare, emphasizing published guidelines by entities like the European Medicines Agency [14]. 

The epidemiology of LSD has been a topic of increasing concern in recent years, especially in regions where cattle farming is a crucial component of the agricultural landscape. Central Thailand, with its sizable cattle population, serves as a prominent example of such a region. The emergence and rapid spread of LSD in this area have not only caused significant economic losses but have also raised questions about the genetic diversity, molecular characteristics, and potential for evolution of LSDV strains within this specific geographic context. In Thailand, LAVs from Kemin (MEVAC, Egypt) and Lumpyvax (MSD, South Africa) have been used since June 2021, following the discovery of LSDV recombinant strains during the first outbreak in March 2021, which was reported in several regions [4,23,24,25]. 

Genetic characterization of LSDV strains is essential for multiple reasons. Firstly, it provides insights into the genetic diversity of the virus, which is crucial for assessing the potential for LSDV to evolve and adapt to different host environments. Understanding the genetic makeup of circulating strains can help in predicting the virus’s behavior and its ability to overcome host immune responses. Secondly, genetic characterization aids in the development and evaluation of effective vaccination strategies. LSD vaccines are essential tools for controlling the disease, and a detailed analysis of the virus’s genome can guide the selection of appropriate vaccine strains and formulations. Furthermore, monitoring genetic changes in the virus can alert researchers and veterinarians to the emergence of vaccine-resistant strains, necessitating timely adjustments in vaccination programs [26]. Thirdly, the genetic characterization of LSDV strains in specific regions, like Lopburi Province, can provide insights into the origin and spread of the virus. This information is valuable for epidemiological studies, helping to trace the sources of outbreaks and understand how the virus is transmitted within and between cattle populations. Lastly, the study of LSDV genomes contributes to the broader scientific knowledge of poxviruses and their molecular biology. Such research can have implications beyond LSD and may aid in understanding related diseases caused by poxviruses in other species.

Additionally, we examined the phylogenetic relationships between the LSDV strains from Lopburi Province and strains from other geographic regions. Whole genome sequencing of LSDV helps researchers identify and characterize various strains and genotypes of the virus. This information is vital for tracking the virus’s evolution, understanding its epidemiology, and developing targeted control measures [17]. As a consequence, it is critical to underline the importance of ongoing surveillance and committed research efforts. These findings are critical for understanding the pattern of LSDV evolution in response to vaccination and, as a result, supporting the development of novel disease management strategies. The primary goal of this study is to track genetic modifications within LSDV following immunization programs in Lopburi Province, Central Thailand, which has a large amount of cattle farming in the country. The detection of such alterations has the potential to promote the development of effective diagnostic and control methods, which could successfully prevent and manage future LSDV epidemics.

## 2. Materials and Methods

### 2.1. Ethics Statement

Cattle owners were interviewed according to the guiding principles for the care and use of research animals. The Institutional Animal Care and Use Committee at KASETSART UNIVERSITY approved the protocol (Project approval number: ACKU64-VET-070). Information about the animals was gathered with the owners’ permission. 

### 2.2. Sample Preparation

Samples were obtained from non-vaccinated beef cattle exhibiting clinical symptoms of LSD that were living in areas that had received vaccinations in Muang Khom and Ko Rang subdistricts of Chai Badan district and Nong Ri subdistricts of Lam Sonthi district in Lopburi Province. Four skin nodule samples were collected from the cattle body using punch biopsy. The skins were homogenized with sterile PBS in a ratio of 1 to 10 and kept at 4 °C until use. The medical histories were recorded individually. The morbidity rate was calculated by dividing the total number of disease cases by the total population in the farms. The mortality rate was calculated by dividing the total number of death cases by the total population in the farms.

### 2.3. Real-Time PCR

DNA was extracted from 10% tissue homogenates using a High Pure PCR Template Preparation Kit (Roche Diagnostics GmbH, Mannheim, Germany) according to the manufacturer’s instructions. The initial screening of an LSD-positive case was carried out using real-time PCR in a 20 μL reaction mixture comprising 200 nM of a primer, 120 nM of a probe, 5 µL of a DNA template, and 10 µL of FastStart Essential DNA Probes Master (Roche Diagnostics GmbH, Mannheim, Germany). CaPV-074F1 5′-AAA ACG GTA TAT GGA ATA GAG TTG GAA-3′ and CaPV-074R1 5′-AAA TGA AAC CAA TGG ATG GGA TA-3′ were used with the minor groove binder (MGB) and the TaqMan probe CaPV-074P1 5′-FAM-TGG CTC ATA GAT TTC CT-MGBNFQ-3′ [27]. The thermal cycler conditions for PCR were run on QuantStudio 5 (Applied Biosystems™, Warrington, UK) followed by a denaturation step at 95 °C for 10 min and then 40 cycles of amplification (15 s at 95 °C and 45 s at 60 °C). The amounts of LSDV DNA were shown as threshold cycle (Ct) values. The standard curve of Ct versus log copy numbers was published previously [28].

### 2.4. LSDV Isolation and Identification

Skin samples that showed a positive Ct value of less than 25 were selected and further processed for LSDV isolation according to the WOAH manual [16]. Briefly, a 10% skin tissue suspension was filtered through 0.45 µm. The processed samples were inoculated onto OA3. Ts cells (CRL-6546™, ATCC^®^, Manassas, VA, USA) were incubated at 37 °C with 5% CO_2_ for four to nine days. Next, the virus isolates were harvested through three freeze–thaw cycles, clarified through centrifugation, and subsequently inoculated onto the OA3. Ts cells were also for the second and third passages. The LSDV isolates were further inoculated onto the Madin-Darby bovine kidney (MDBK) cell line (CCL-22™, ATCC^®^, Manassas, VA, USA) at 37 °C with 5% CO_2_ for the further passages and CPE was observed daily for four to nine days. The cell was observed for the appearance of a cytopathic effect (CPE), and the virus was collected through three freeze–thaw cycles, aliquoted, and stored at −80 °C. Real-time PCR was used to analyze the amount of virus in every passage. Virus titration or TCID_50_/mL was determined by a TCID50 assay using MDBK cells.

### 2.5. Whole Genome Sequencing (WGS) for LSDV 

LSDV genomic DNA was extracted from virus isolates using the DNeasy blood and tissue kit (Qiagen, Hilden, Germany), according to the manufacturer’s instructions. Methylated bovine DNA contaminants in LSDV DNA samples were removed using the NEBNext^®^ Microbiome DNA Enrichment kit (New England Biolabs, Ipswich, MA, USA) according to the manufacturer’s instructions. The DNA quality was then checked by measuring the absorbance ratio at 260/280 and 260/230 nm with a Nanodrop spectrophotometer (Thermo Fisher Scientific, Waltham, MA, USA). DNA concentration was measured with a Qubit fluorometer with a dsDNA High Sensitivity assay (Invitrogen, Carlsbad, CA, USA). The DNA was stored at −20 °C until used in the next step. The DNA library was constructed using the Nextera XT DNA library preparation kit. A MiSeq benchtop sequencer (Illumina, San Diego, CA, USA) and a MiSeq reagent kit version 3 were used for the sequencing (600 cycles) or version 2 (500 cycles) (Illumina, San Diego, CA, USA) paired-end sequencing. Using the FastQC software (https://www.bioinformatics.babraham.ac.uk/projects/fastqc/ (accessed on 6 December 2022)), raw data quality was evaluated. A BBDuk Trimmer was used to trim the readings based on length (>20 bp) and quality (Q score > 30). Geneious Prime software version 2021.2.2 (Biomatters Ltd., Auckland, New Zealand) was used for genome assembly and annotation. Using the SPAdes assembler version 3.15.2, the trimmed reads were de novo assembled into contigs and aligned to the LSDV/KM/Taiwan/2020 (OL752713) and LSDV/Thailand/Yasothon/2021 (OM033705) reference genomes.

### 2.6. DNA Sequencing, ORF023

Virus isolates were extracted using the High Pure PCR Template Preparation Kit (Roche Diagnostics GmbH, Mannheim, Germany). DNA amplification and sequencing targeting 554 bp covering the ORF023 gene and flanked UTR were conducted with specific primers using a Q5^®^ Hot Start High-Fidelity 2× Master Mix (New England Biolabs, Ipswich, MA, USA) according to the manufacturer’s instructions (Table S1). Following the amplification, the PCR products were analyzed through gel electrophoresis and purified using the MinElute PCR Purification Kit (Qiagen, Hilden, Germany). DNA sequencing was then performed using the BigDye terminator v3.1 Cycle Sequencing Kit (Applied Biosystems™, Waltham, MA, USA), following the manufacturer’s instructions. The analysis of the DNA sequences was performed using the ABI 3500 genetic analyzer (Applied Biosystems™, Waltham, MA, USA). 

### 2.7. Nucleotide Mutation and Phylogenetic Analysis

MAFFT alignment in Geneious Prime software was used for aligning the studied sequences with CaPV strains from GenBank [29]. A complete genome phylogenetic tree was built using the Maximum Likelihood (ML) method with the GTR + G + I model and 1000 bootstraps by MEGA 11. To evaluate mutation, four sequences were aligned compared with LSDV/Thailand/Yasothon/2021 (YST strain) using MAFFT pairwise alignment in Geneious Prime software. The annotation from the Yasothon strain was transferred using the program. The SNPs position was located using variations/SNPs tools. Amino acid alterations were shown through the translation function. To ensure the mutation did not occur from human or technical errors during the analysis, the depth of coverage for all mutation locations was recorded.

## 3. Results

### 3.1. Animal and Sample Data

**Figure 1:**
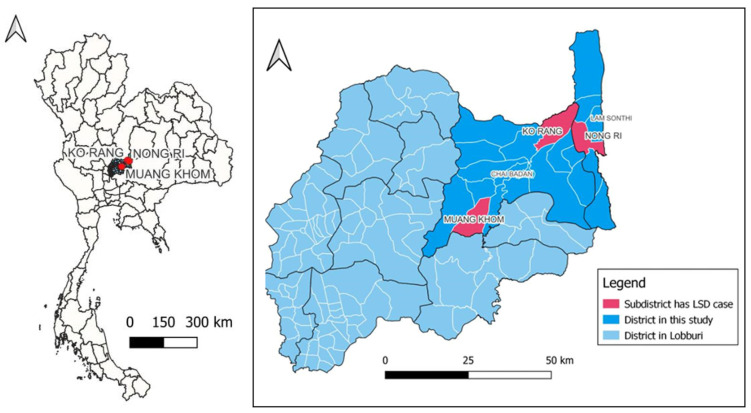
Geographical mapping of studied locations at Muang Khom and Ko Rang subdistricts of Chai Badan district, and Nong Ri subdistrict of Lam Sonthi district in Lopburi Province.

In total, twelve samples were obtained during 13 months from Lopburi Province during September 2021 to September 2022. Four samples selected for virus isolation and WGS were collected between September 2021 and June 2022 when using the live-attenuated vaccine in Lopburi Province from June 2021. The history of four non-vaccinated beef cattle indicated that skin lesions were collected from 2–5-month-old cattle that exhibited clinical signs of LSD including skin nodules, fever, and lymph node enlargement. The morbidity and mortality rates are presented in Table 1. These four animals came from three different farms in Muang Khom and Ko Rang subdistricts of Chai Badan district and Nong Ri subdistricts of Lam Sonthi district in Lopburi Province, Thailand (Figure 1).

### 3.2. Virus Detection

All of the initial four skin samples had Ct values of LSDV074 *p32* ranging from 19.39 to 22.51 (Table 1). All four skin samples were cultured 3–7 passages further to increase viral particles for WGS. All of them presented a cytopathic effect (CPE) as groups of aggregated LSDV infected cells at 3 days post infection of the second passage and were positive based on the real-time PCR *p32* gene. The Ct values and TCID_50_/mL of the virus isolates from each passage are shown in Table S2. The Ct comparison with the viral genome copy number was not included in this study as it was previously published [28].

### 3.3. Whole Genome Sequencing and Phylogenetic Analysis

**Figure 2:**
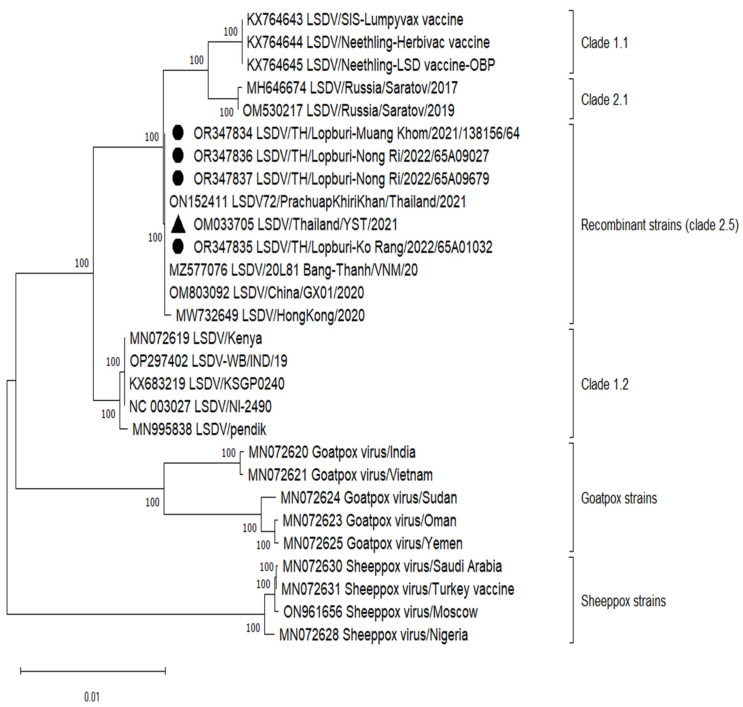
Phylogenetic analysis of whole genome lumpy skin disease virus in this study with 24 Capripoxvirus reference strains. The tree was constructed using MEGA 11 with the Maximum Likelihood method, GTR+G+I model, and 1000 bootstraps. The sequences obtained from this study are OR347834, OR347835, OR347836, and OR347837 indicated by solid black circles. The reference Thai strain used for mutation analysis is OM033705 indicated by solid black triangle. Clade was assigned according to a previous study [30].

Table 2 displays comprehensive genomic information, including the overall read count, coverage depth, and nucleotide identities in comparison to the Yasothon strain. The entire genomic sequences of four LSDVs have been submitted to the GenBank repository (accession numbers: OR347834-OR347837). These genome sequences exhibit lengths ranging from 150,854 to 150,857 nucleotides, featuring a total of 156 predicted protein-coding genes. The LSDVs in this study were clustered in recombinant LSDV strains (clade 2.5), same as LSDV/Yasothon(YST)/2021 (Figure 2). All mutations were confirmed by Sanger sequencing of full-length ORF023 and the UTR. However, Muang Khom and Ko Rang strains started mutations at passage 4 and 3, respectively. The genetic mutation could be observed from the overlapping of the base peaks, and the mutant population increased to become the major population in passage 5 (Muang Khom) and passage 4 (Ko Rang). Interestingly, Nong Ri strains had mutation at the first passage. 

### 3.4. Mutation Analysis

**Figure 3:**
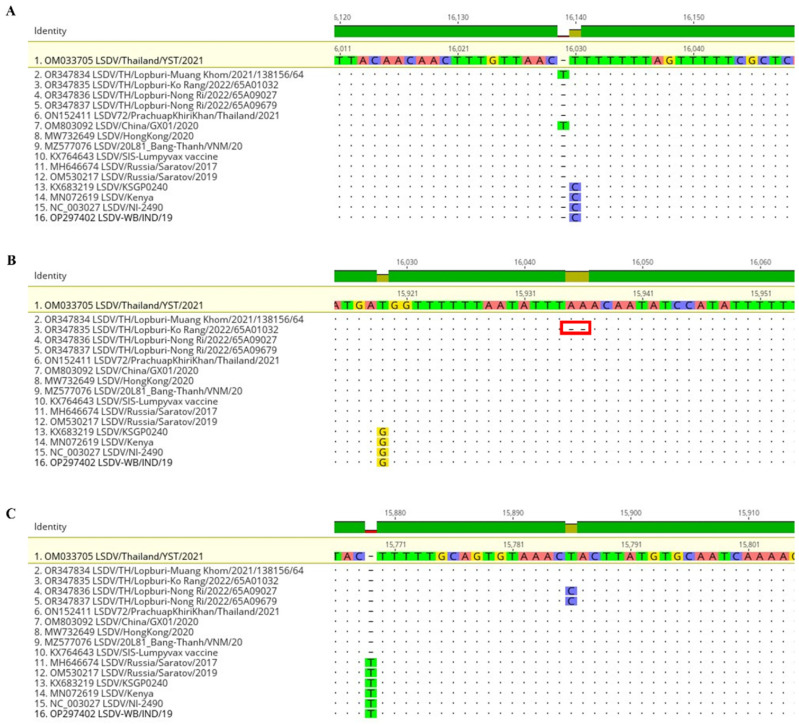
Alignment of four LSDVs in this study with 12 reference strains by setting LSDV/Thailand/YST/2021 as a reference. (**A**) Insertion of T at 16,030 (ORF023) of Muang Khom strain. (**B**) Deletion of AA at 15,935–15,936 (ORF023) of Ko Rang strain. (**C**) Transition T to C at 15,786 (UTR between ORF022 and ORF023) of Nong Ri strains. The analysis was performed through Geneious software ^®^.

Compared to the LSDV/Thailand/YST/2021 (OM033705) strain, four samples from Lopburi presented one mutated gene. The nucleotide position 16,030 of the Muang Khom strain had a T insertion mutation causing 64 amino acids deletion from 73 to 9 amino acids in ORF023 encoding hypothetical protein (Table 2, Figure 3A and Figure S1A). The nucleotide position 15,935–15,936 of the Ko Rang strain showed two A deletions mutation causing amino acid change from Leu Asn Ile to Lys Tyr and deletion of 32 amino acids from 73 to 41 amino acids in ORF023 (Table 2, Figure 3B and Figure S1B). Nong Ri strains showed a transition mutation at nucleotide position 15,786 from T to C causing no amino acid change in the untranslated region between ORF022 and ORF023 (Table 2, Figure 3C and Figure S1C). In total, the only one from 156 genes was a maximum number of mutation genes of the whole genome (less than 1%). A coverage depth of all mutation points ranged between 388.1 to 523.8 (Table 2).

The alignment of four LSDVs from Lopburi Province with 12 reference strains showed that an insertion of T at position 16,030 of LSDV/2021 was previously presented in LSDV China/GX01/2020 (OM803092) (Figure 3A), while the deletion of two A at the position of 15,935–15,936 and the transition mutation at the position of 15,786 of LSDV/2022 were unique and have never been reported (Figure 3B–C). Notably, original field strains such as KSGP 0240, Kenya, and NI-2490 are C distinct from vaccine and recombinant strains that displayed T at 16,030. At 15,919 G/T variance, filed strains can be distinguished from vaccine and recombinant strains. T was deleted at 15,770 in the vaccine and recombinant strains except for clade 2.1 Russia/Saratov/2017 and 2019.

## 4. Discussion

The genomic analysis of LSDV strains circulating among beef cattle in Lopburi Province, Central Thailand, during 2021–2022, has yielded critical insights into the genetic diversity and molecular characteristics of this economically significant pathogen. Our research has unveiled several notable aspects of the LSDV strains in this region, which hold significance not only for local veterinary authorities and cattle farmers but also for the broader scientific community dedicated to the study of poxviruses and infectious diseases. By elucidating the genomic makeup of LSDV strains, our study addresses the pressing need for a deeper understanding of the pathogen’s dynamics. The implications of our findings extend beyond the immediate context of Lopburi Province, having broader applications in enhancing global knowledge about LSDV and related poxviruses. This is particularly relevant in the current era of interconnectedness, where diseases can quickly transcend geographical boundaries. It is essential to recognize the potential for LAVs to catalyze recombination events between vaccine-derived and wildtype viral strains, potentially leading to the emergence of more virulent progeny [17,31,32]. Historical examples, such as the LSDVs in South Africa during the 1990s, highlight instances of potent strains, both virulent and vaccine-associated, characterized by multiple single nucleotide polymorphisms (SNPs) compared to the attenuated vaccine strains [33]. This underscores the need for consistent surveillance of the genetic architecture of the entire viral genome, particularly in genes associated with virulence and host specificity.

In a previous study, our research identified mutations in LSDV strains from five provinces in different regions of Thailand including Chiang Mai (North), Khon Kaen (Northeast), Nakhon Pathom (Central), Prachuap Khiri Khan (West), and Trang (South) specifically in ORF032, ORF094, ORF133, ORF144, and ORF148 [26]. Notably, the mutations in ORF023 with amino acid deletions and in the untranslated region (UTR) identified in our current study have not been previously reported in the country. In this study, the ORF023 gene of the virus culture from Chai Badan samples was intact during the first two passages but started showing mutations in the third and fourth passages, causing several amino acids’ deletion. This suggested that the protein translated by this gene is unlikely to be important for growth in MDBK cultured cells due to the virus still presenting the CPE and being a large amount of virus (Ct less than 25). However, further studies need to be performed to see what effect the absence of this protein has on the host, such as whether the animal can develop immunity or not. This is because vaccine production requires multiple passages of the virus, which may cause this gene to disappear. In contrast, the samples from Lam Sonthi district showed mutation at the first passage, indicating the unique virus character in the area. From preliminary experiments by the NIAH, the LSDV/Roi Et/991.2/2021 recombinant strain at the 30th passage had very few mutations. The mutation found only one base at the UTR position between ORF022 and ORF023 (unpublished). Another study in India found that the live-attenuated vaccine produced from a field virus at the 50th passage in Vero cells had significant deletion mutations in the inverted terminal repeat region (ITR) that has never been reported in other Neethling strains [22]. The vaccination, on the other hand, can stimulate HMI and CMI responses and provides complete protection against a virulent LSDV challenge. Thus, it is important to select the proper strain for vaccine production.

A plausible determinant for the disparity in genetic alteration among LSDV strains may be influenced by the varying sizes of bovine populations in different regions. The variation in genetic alteration could be attributed to the fact that the five regions in Thailand mentioned previously had a smaller bovine population in comparison to the current study area. The association between population size and genetic variation underscores the need to consider demographic factors when analyzing the genetic landscape of LSDV strains, as larger populations may facilitate increased opportunities for genetic diversity and alterations to occur [26,34]. Chai Badan exhibited a notably larger beef cattle population, numbering approximately 9587 heads compared to Lam Sonthi’s 5898 heads. Notably, despite the synchronicity of the initial LSD outbreaks within the Chai Badan and Lam Sonthi districts in August 2022, the genetic analysis of the implicated LSDVs suggests that these occurrences likely originated from distinct sources. In the case of Chai Badan, the mutation analysis pinpointed ORF023 as a locus of variation, while in Lam Sonthi, the variance was localized within the UTR region. The ORF023 mutation previously identified in the LSDV/PrachuapKhiriKhan/2021 strain, an isolate associated with an earlier outbreak in the western region [23], did not result in modifications in protein length. This raises intriguing questions about the role of this mutation and its adaptive significance in the specific agro-environmental context of Chai Badan. Additionally, the presence of T insertion at nt 16,030 of Muang Khom strain passage 5 was identical to the LSDV Chinese strain in 2020 (OM803092) [18]. However, the passage number of the Chinese strain was not available. Conversely, the uniform circulating virus profile observed across the cattle population in Lam Sonthi suggests a lack of introduction of novel cattle, aligning with historical animal records.

The observed morbidity and mortality rates within the context of the three examined farms were slightly above the previous reports, i.e., below 40% and 3%, respectively [23,25]. This suggests that the LSDV/Lopburi strains may substantially influence the pathogenic potential of the causative agent. However, it is essential to exercise caution in drawing definitive conclusions based solely on these rates. A more comprehensive evaluation through in vivo [35] and in vitro experiments is warranted to conclusively assess the impact of these mutations on virulence and host interactions [36]. Initially, the appearance of CPE was similar to our previous study [4].

The classification system proposed by Breman et al. [30] has placed the LSDV/PrachuapKhiriKhan (PKK) strains within clade 2.5, alongside some Vietnamese, Chinese, and Yasothon strains, which were further divided into clade 2.5.1. It is noteworthy that the Yasothon sample was collected in April 2021, predating the PKK sample collected in September 2021. This discrepancy in cladding may be attributed to the release of the WGS of the Yasothon strain occurring after that of the PKK strain. Such nuances in clade assignments underscore the importance of continuous updates in genomic databases to accurately reflect the evolutionary relationships among LSDV strains.

It is encouraging to note that our analysis did not reveal the presence of other clades, especially the field strains of cluster 1.2 (Indian subcontinent), within the country. This suggests that the preventive and control measures implemented by the Department of Livestock Development (DLD) have been effective in containing the spread of these particular strains. However, continued vigilance and surveillance are imperative to ensure the ongoing success of these control measures. Drawing from the clinical manifestations observed and the phylogenetic analysis, it is plausible to assert that the LAVs continue to confer protection against the mutated strains. This aligns with previous documentation that has indicated a marked reduction in LSDV outbreak instances attributed to recombinant variants following the adoption of LAVs within the region [4]. The effectiveness of LAVs in providing immunity against a spectrum of LSDV strains underscores their critical role in disease control efforts [14,37]. The effects of LSDV outbreaks on society and economy are not only for cattle farmers and veterinary authorities, but the entire community by causing negative impact on trade and jobs. In Ethiopia, most economic losses were attributed to mortality (USD 1000) and milk loss (USD 120) [38]. The northeastern region of Thailand also experiences large financial losses in the dairy and beef cattle industries. During the outbreak period, it was estimated to have cost THB 2,413,000 (USD 68,943) in economic losses. Dairy farmers lost between 8.23 and 9.96 tons of milk [39]. The discovery of particular mutations and their possible impact on pathogenicity emphasizes the necessity of an all-encompassing strategy for disease control that takes into account the community’s financial stability in addition to the health of the cattle population. This more comprehensive viewpoint emphasizes how closely related local economies, agriculture, and animal health are. Moreover, the genomic analysis provides a foundation for developing targeted interventions and refining vaccination strategies. The precision in vaccine development can enhance efficacy and contribute to the sustainable control of LSDV. By leveraging genomic information, the scientific community can contribute to the tools available for combating LSDV and similar poxviruses.

It is essential to acknowledge the limitations of our study. While we have identified genetic variations, further research is needed to elucidate the functional consequences of these mutations. Experimental studies, including in vitro and in vivo assays, can provide insights into how these genetic changes influence the virus’s behavior, virulence, and host interactions. Additionally, ongoing surveillance of LSDV strains in Lopburi Province and other regions is crucial to monitor the spread and evolution of the virus. Long-term genetic monitoring can help identify emerging strains and assess their impact on disease control efforts and vaccine efficacy.

## 5. Conclusions

The four LSDVs circulated in Lopburi in 2021–2022 were recombinant strains of clade 2.5, same as the Yasothon/2021 strain. We identified mutations of ORF023 with significant amino acid deletion in two isolates from Chai Badan district and of UTR in two samples from Lam Sothi district. These findings have never been reported. The unique mutations identified in Lopburi strains underscore the dynamic nature of LSDV genomes and the need for ongoing genetic surveillance to understand and respond effectively to emerging strains. Additionally, our study contributes to the broader scientific knowledge of LSDV genetic diversity, which is essential for developing effective vaccines and control strategies for this economically significant cattle disease. Further research is warranted to explore the functional consequences of these mutations and their implications for LSDV virulence and host interactions.

## Figures and Tables

**Table 1:** Details of the samples.

No.	Sample Received Date	Animal	Age (Months)	Morbidity Rate (%)	Mortality Rate (%)	Subdistrict	District	Initial Ct	Passage for WGS
1	28 September 2021	Beef cattle	2	4	0	Muang Khom	Chai Badan	19.4	5
2	15 February 2022	Beef cattle	2	50	0	Ko Rang	Chai Badan	19.4	6
3	10 June 2022	Beef cattle	5	11.76	5.88	Nong Ri	Lam Sonthi	20.9	4
4	21 June 2022	Beef cattle	5	11.76	5.88	Nong Ri	Lam Sonthi	22.5	3

**Table 2:** Whole genome sequencing analysis compared to LSDV/Thailand/Yasothon/2021 (YST).

No.	Number of Reads	Number of Trimmed Reads (Q30/L20)	Size of Largest Contig or Scaffold (bp)	Number of Trimmed Reads *	Mean of Coverage Depth *	Genome Size (bp)	% Identity to YST/2021	Number of Differences in Nucleotide	Affected ORF (Protein)	SNPs (Position)	Mutation Type	Amino Acid Change	GenBank Accession No.
1	9,048,280	7,540,224	146,141	399,550 399,549	415.6 414.7	150,857	99.9993	1	023 (hypothetical protein)	- → T (16,030)	Insertion	V → S*stop (del 64 aa)	OR347834
2	26,229,520	22,445,168	145,884	441,854 537,976	335.6 523.8	150,854	99.9987	2	023 (hypothetical protein)	AA → -- (15935–15936)	Deletion	LNI → KY*stop (del 32 aa)	OR347835
3	9,278,280	7,587,054	145,886	377,662 377,661	388.5 388.1	150,856	99.9993	1	UTR	T → C (15,786)	Transition	-	OR347836
4	15,513,540	13,656,538	145,886	333,454 333,454	389.8 389.3	150,856	99.9993	1	UTR	T → C (15.786)	Transition	-	OR347837

* Mapped to OM033705 and OL752713, respectively. Q30/L20 = The reads were trimmed with a minimum quality score of 30 and a minimum of 20 bases in length. ORF = Open reading frame. UTR = Untranslated region. SNPs = Single nucleotide polymorphisms. - → T = T insertion, AA → -- = two A deletion.

## Data Availability

Accession numbers OR347834-OR347837 are available in GenBank.

## Supplementary Materials

The following supporting information can be downloaded at: https://www.mdpi.com/article/10.3390/vetsci11010010/s1, Figure S1: Nucleotide alignments of each LSDV strain compared to Yasothon/2021.; Table S1: Details of PCR primers for sequencing ORF 023 of LSDV; Table S2: Quantification of LSDV isolates in OA3. Ts or MDBK cells.
