# Supplementary material for: Complete Genomic Characterization of Lumpy Skin Disease Virus Isolates from Beef Cattle in Lopburi Province, Central Thailand, during 2021–2022
Source: Vet Sci. 2023 Dec 22;11(1):10. doi: 10.3390/vetsci11010010 (PMC10818611; doi:10.3390/vetsci11010010)
Supplement: Supplementary file 1 [file vetsci-11-00010-s001.zip › vetsci-2658458-supplementary.pdf]

Table S1. Details of PCR primers for sequencing ORF 023 of LSDV

Primer name	Primer sequence (5'-3')	T_m (°C)	Annealing temp (°C)	Amplicon size (bp)	Start (5')	End (3')	Primer length (bp)	%GC
L23F2	TTTTGACGATTTTATACATCCTTCATAG	60	61	554	15,711	15,738	28	29
L23R2	CATTCATCAACGATGTTCATTGG	62			16,238	16,265	23	39

Table S2. Quantification of LSDV isolates in OA3. Ts or MDBK cells

NO.	Initial Ct (*)	P1			P2			P3			P4			P5			P6			P.7		
		OA3.Ts		TCID50	OA3.Ts		TCID50	OA3.Ts		TCID50	MDBK		TCID50	MDBK		TCID50	MDBK		TCID50	MDBK		TCID50
		CPE	Ct (*)		CPE	Ct (*)		CPE	Ct (*)		CPE	Ct (*)		CPE	Ct (*)		CPE	Ct (*)		CPE	Ct (*)	
1	19.4 (10.8)	+	UND	UND	+	23.8 (6.6)	UND	+	25.5 (6.1)	$10^{2.63}$	+	22.7 (6.8)	$10^{2.80}$	+	23.7 (6.6)	$10^{4.80}$	+	18.4 (8.0)	$10^{5.73}$	+	17.6 (8.2)	$10^{6.10}$
2	19.4 (10.8)	+	31.1 (4.6)	UND	+	23.4 (6.6)	$10^{2.63}$	+	25.2 (6.2)	$10^{3.97}$	+	23.4 (6.7)	$10^{3.80}$	+	19.7 (7.6)	$10^{6.80}$	+	14.8 (9.0)	$10^{4.80}$	+	18.6 (7.9)	$10^{5.97}$
3	20.9 (7.3)	+	34.84 (3.6)	UND	+	30.8 (4.7)	UND	+	28.2 (5.4)	UND	+	29.4 (5.1)	UND	+	24.1 (6.5)	$10^{3.80}$	NA	NA	NA	NA	NA	NA
4	22.5 (6.8)	+	UND	UND	+	24.2 (6.4)	UND	+/-	25.1 (6.2)	UND	+	23.5 (6.6)	UND	+	24.6 (6.3)	$10^{3.80}$	+	21.9 (7.1)	$10^{4.80}$	+	20.9 (7.3)	$10^{4.97}$

*($\log_{10}$copies/ul)

UND: undetected

NA: not applicable

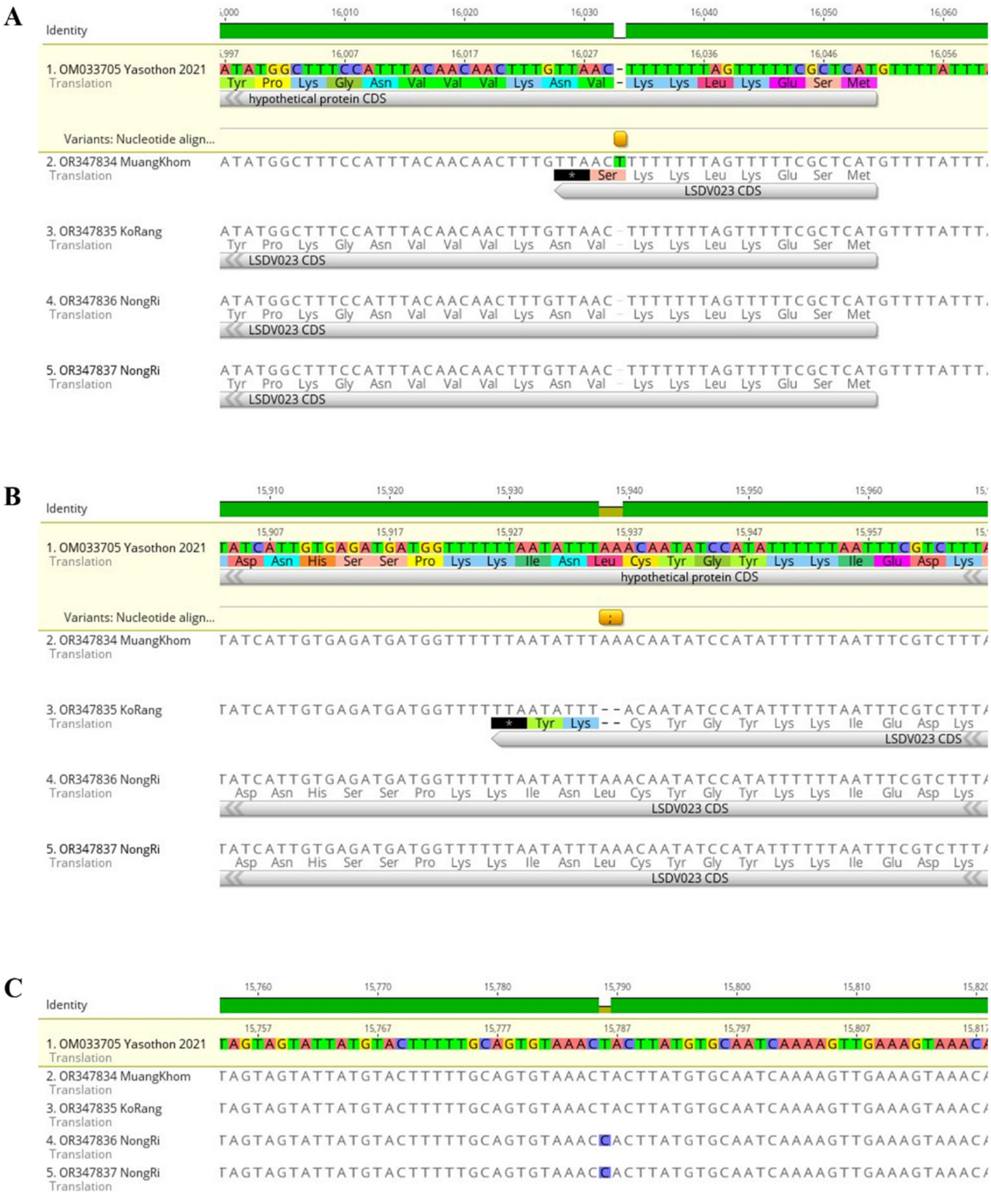

Figure S1: Nucleotide alignments of each LSDV strain compared to Yasothon/2021. A) LSDV 138156/64/Lopburi/2021 strain had a T insertion mutation at nucleotide position 16,030 causing 64 amino acids deletion from 73 to 9 amino acids in ORF023 encoding hypothetical protein. B) LSDV 65A01032 Lopburi/2022 strain had two A deletions mutation at nucleotide position 15,935-15,936 causing amino acid change from Leu Asn Ile to Lys Tyr and deletion 32 amino acids from 73 to 41 amino acids in ORF023. C) LSDV 65A09027 and 65A09679 Lopburi/2022 strains had a transition mutation from T to C at nucleotide position 15,786 causing no amino acid change in untranslated region between ORF022 and ORF023.
